# Supplementary figures and images for: Infection with multiple HIV-1 founder variants is associated with lower viral replicative capacity, faster CD4+ T cell decline and increased immune activation during acute infection
Source: PLoS Pathog. 2020 Sep 4;16(9):e1008853. doi: 10.1371/journal.ppat.1008853 (PMC7498102; doi:10.1371/journal.ppat.1008853)

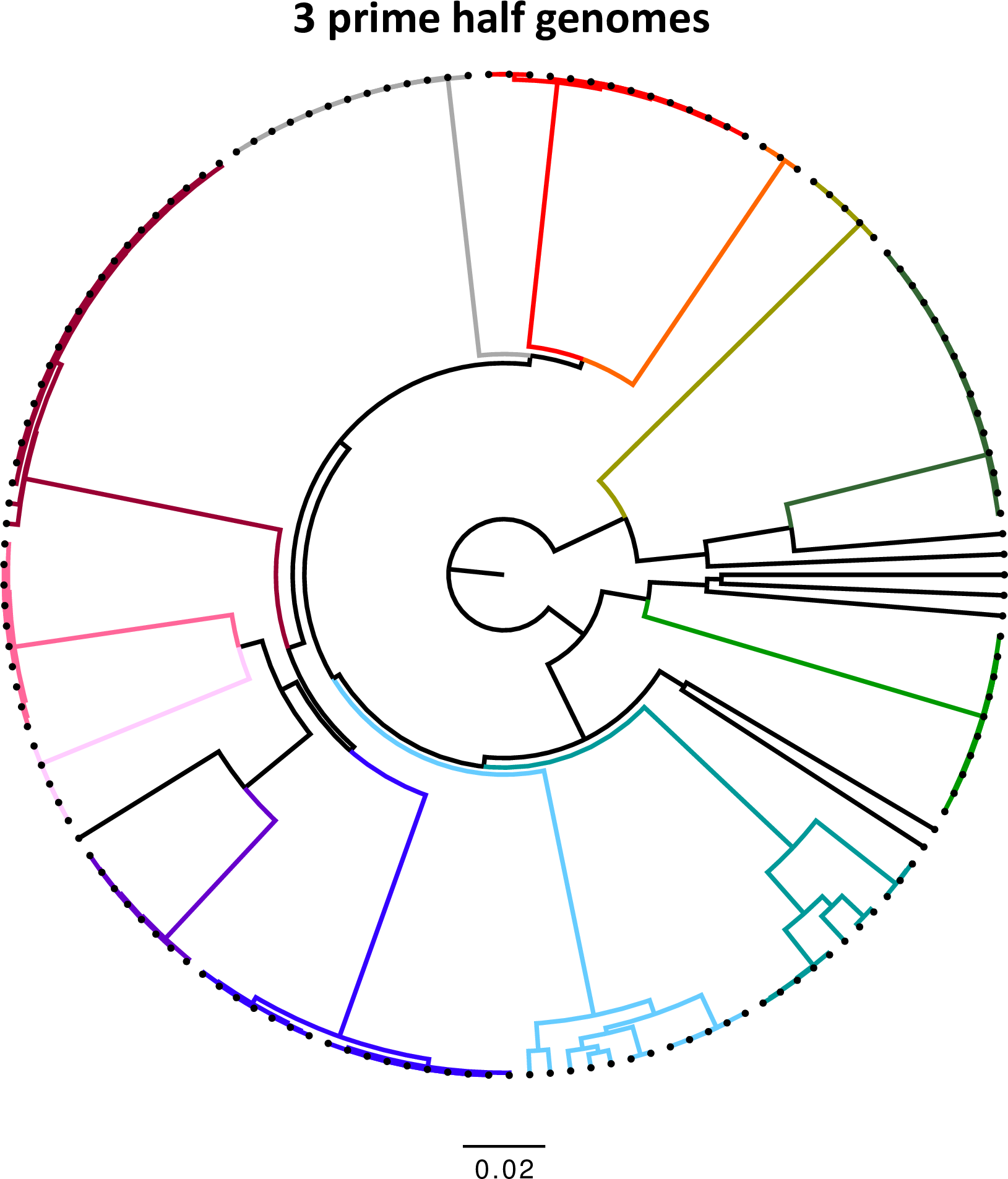

Supplement: S1 Fig — Phylogenetic tree based on A) 3 prime half genome nucleotide sequences from 13 participants. Sequences on branches of the same colour were derived from the same individual, while sequences in black correspond to subtype references. (TIF) [file ppat.1008853.s001.tif]

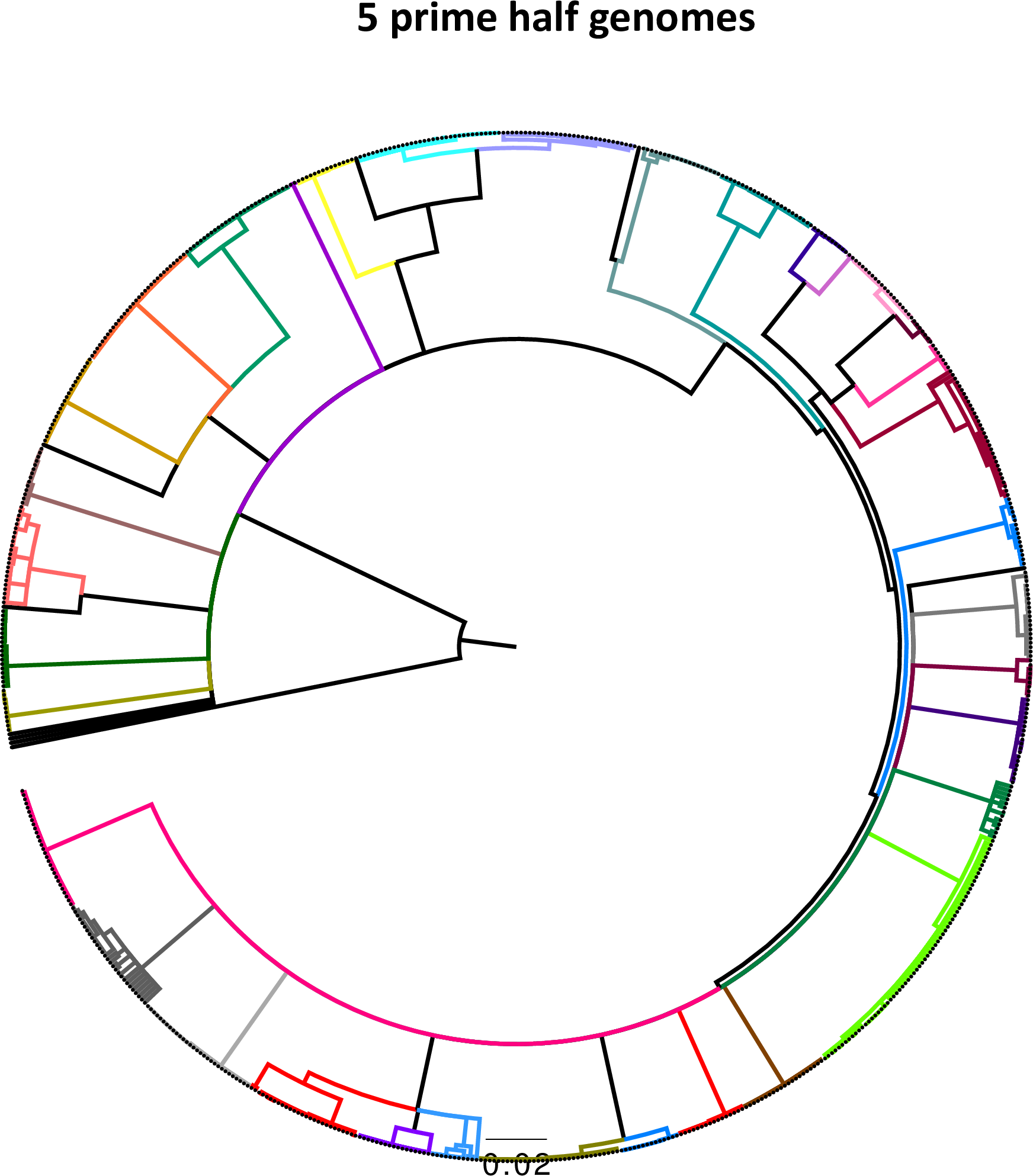

Supplement: S2 Fig — Sequences on branches of the same colour were derived from the same individual, while sequences in black correspond to subtype references. (TIF) [file ppat.1008853.s002.tif]

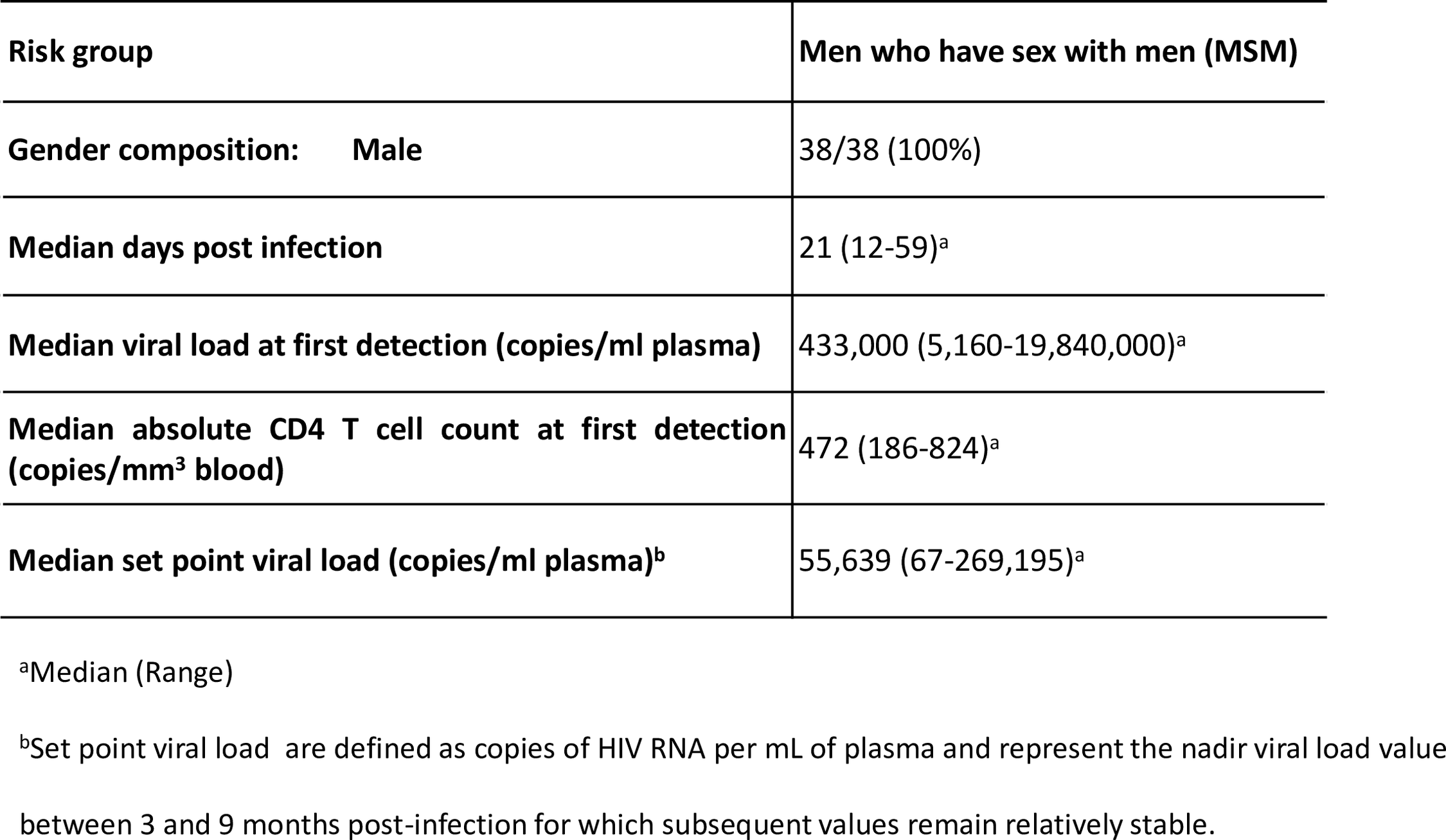

Supplement: S1 Table — (TIF) [file ppat.1008853.s003.tif]

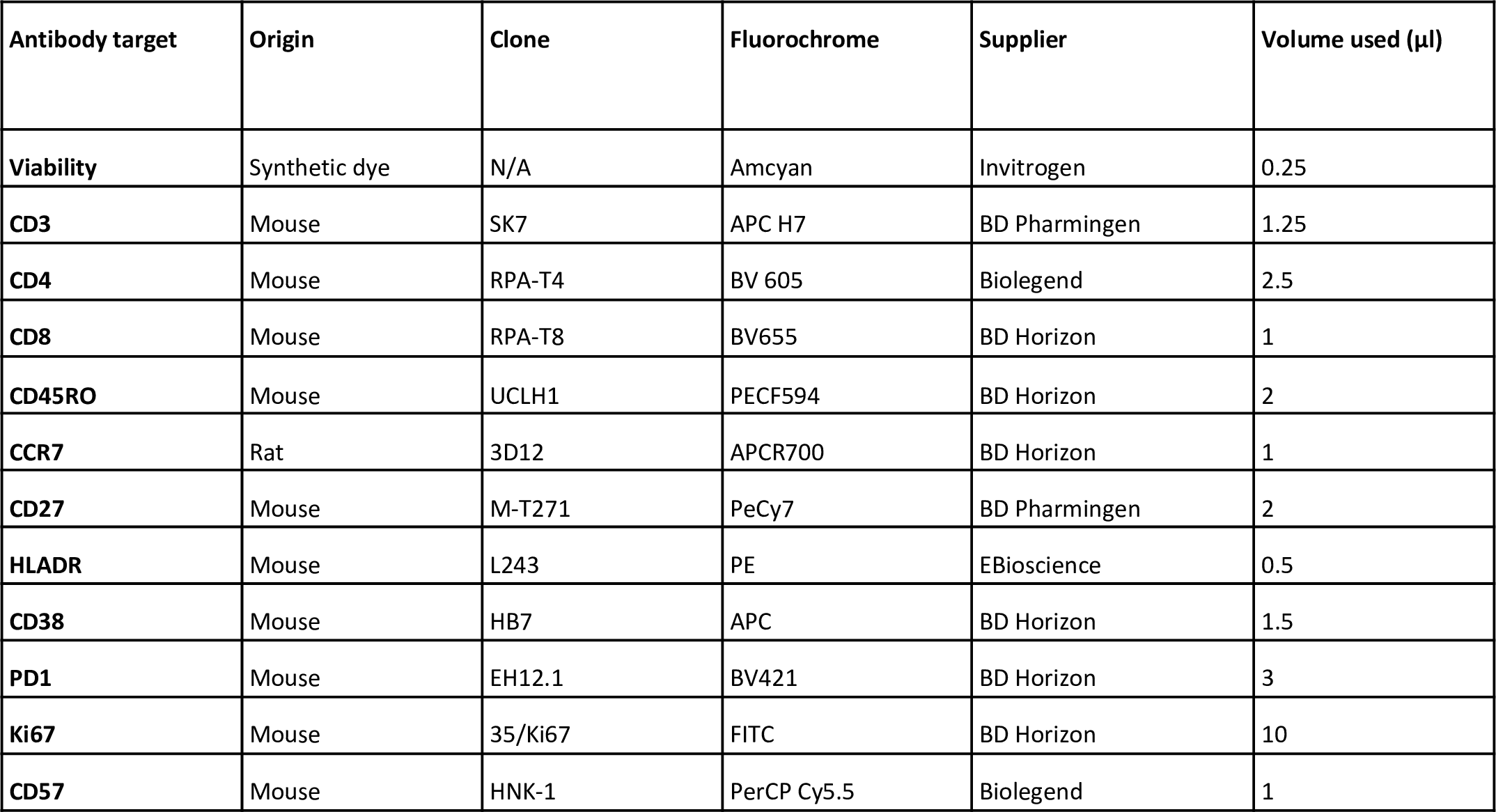

Supplement: S2 Table — (TIF) [file ppat.1008853.s004.tif]

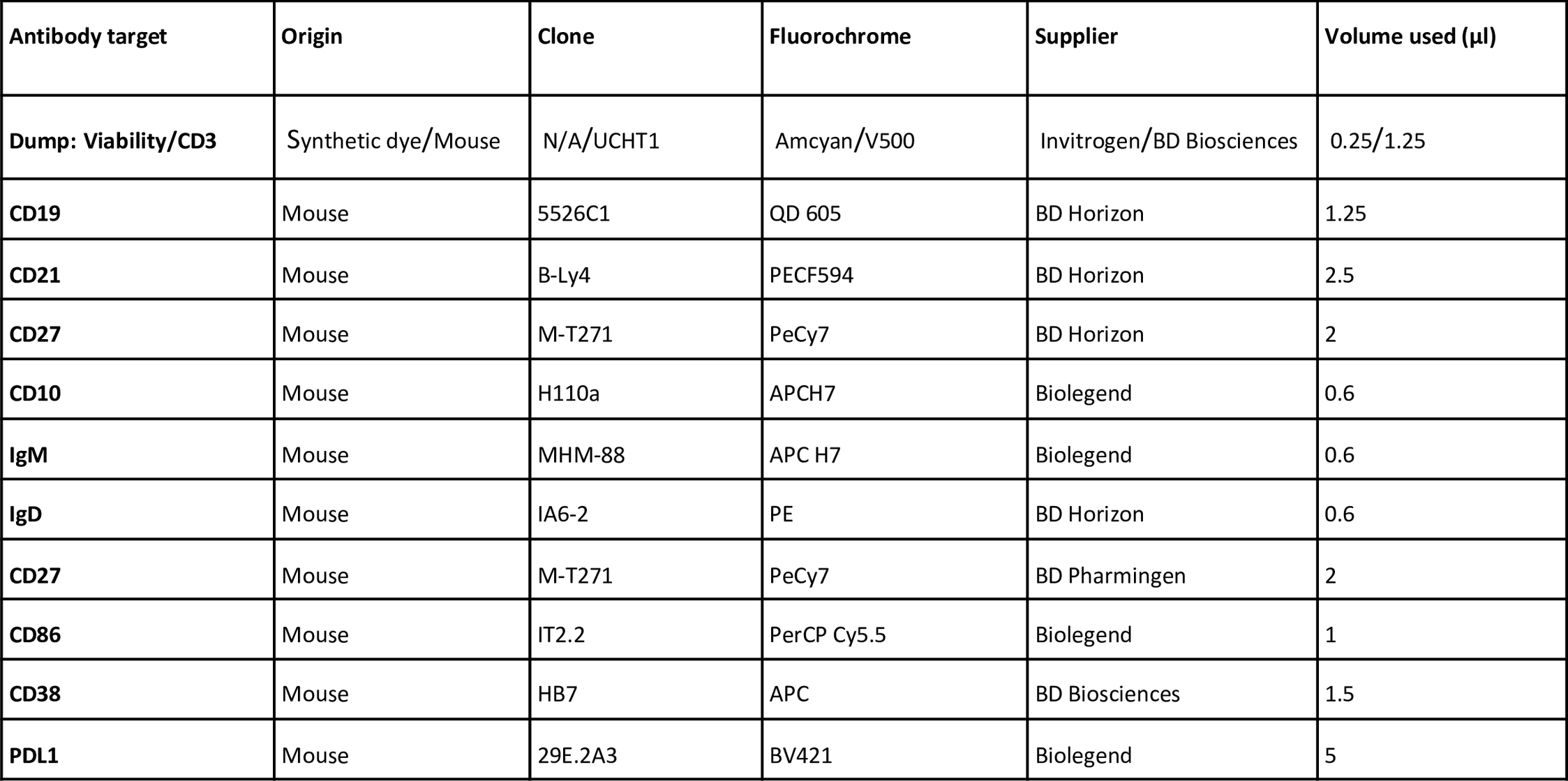

Supplement: S3 Table — (TIF) [file ppat.1008853.s005.tif]
