# Supplementary material for: Infection with multiple HIV-1 founder variants is associated with lower viral replicative capacity, faster CD4+ T cell decline and increased immune activation during acute infection
Source: PLoS Pathog. 2020 Sep 4;16(9):e1008853. doi: 10.1371/journal.ppat.1008853 (PMC7498102; doi:10.1371/journal.ppat.1008853)
Supplement: Attachment — Submitted filename: Reviewer comments - 06July2020 2.docx [file ppat.1008853.s006.docx]

**To the Editors, PLOS Pathogens**

**Re: Responses to Reviewers’ comments on " Infection with multiple HIV-1 founder variants is associated with lower viral replicative capacity, faster CD4+ T cell decline and increased immune activation during acute infection" by Macharia et al.**

We would like to thank the **editors** at PLOS Pathogens and the **reviewers** for their careful reading and valuable commentary on our manuscript “Infection with multiple HIV-1 founder variants is associated with lower viral replicative capacity, faster CD4+ T cell decline and increased immune activation during acute infection.” We found that all the comments from the reviewers’ and editors’ strengthened the interpretation and presentation of our data. The reviewers raised several issues that needed clarification additional analysis, both of which have been beneficial to the understanding and interpretation of the data presented in this manuscript. We are now resubmitting a significantly revised version that takes into account the reviewers’ comments as detailed below.

**Point-by-Point Response to Reviewer #1’s remarks:**

1. *Reviewer’s remarks: A larger percentage of acutely infected MSM individuals harbour multiple founder strains. This is not a novel finding. Indeed, the authors cite the publication from Li et. al. although they fail to adequately acknowledge that this is not a new finding. Authors should make a more persuasive argument why there finding is unique. They demonstrate that individuals with multiple as compared to single founders generally faster disease progression. They found that multiple as compared to single had faster CD4 decline although not higher virus level. This finding is also not novel, and it has also been previously demonstrated in different cohorts.*

During HIV transmission among men who have sex with men (MSM), infection with multiple transmitted/founder variants has been previously reported, but findings are conflicting, with some studies demonstrating establishment of infection by single founders only 60 percent of the time, while others report similar rates among MSM and heterosexuals (1-7)**.** These were studies of subtype B infected cohorts in the US and none included subtype A infection. Worse disease outcome has also been shown in patients infected with multiple transmitted/founder variants with some cohorts observing higher SPVL but not faster CD4 T cell decline (1, 3).

We have had a unique opportunity to now validate this phenomenon in one of the first well described MSM cohorts in East Africa (8, 9). This is the first time a rural East African MSM cohort, infected with Clade A viruses has been studied, and where samples have been available from very early infection (all earlier than 60 days post infection and median 21 days) compared to 143 and 11-170 days post infection in Janes et al and Chantillon et al respectively (1, 3). This early sampling enables the characterisation of transmitted/founder variants before early diversification. In addition, some studies that demonstrated an association between multivariant infection and higher viral loads but not CD4 T cell counts included breakthrough infection in vaccine trial participants (3, 6), unlike our study which is in unvaccinated MSM. Moreover, these studies did not investigate whether the in-vitro replicative capacity of transmitted/founder variants that establish multivariant infection differed from that of variants that are transmitted as single variants.

Our findings from an early infection subtype A cohort demonstrates that multivariant infection in MSM was associated with a less stringent transmission bottleneck that allowed for less fit variants to establish infection and was associated with worse disease outcome and immune perturbations. While both the aspect of a reduced bottleneck in MSM versus heterosexual transmission, as well as an association of multiplicity of infection with disease progression have previously been reported on, albeit with a wide range of observations, this study is novel in that it investigates replication capacities of HIV variants from single versus multivariant infection and reports a more rapid decline in CD4+ T cells, a known marker of disease progression, in individuals infected with more than one variant. In addition, we reiterate the need for treatment of existing STIs as a tool in the prevention and slowing of disease in vulnerable populations. The manuscript has been amended and highlighted in track changes to reflect the comments above.

2. *Reviewer’s remarks: They examined the replication capacity conferred by the gag gene among the multi- and single founder cases. In figure 3, they find that among the multiple founder cases, the minority strain’s gag often confers higher replicative capacity.*
   a. *Fig. 3B appears to hint at this finding although, it has not been adequately analysed. I would suggest that they do a matched pairwise comparison between the major variant and an average or median of the minority variants among the 7 analysed cases.*

We thank the reviewer for highlighting this crucial point. As suggested by the reviewer, we have now performed a matched pairwise comparison between the major variant and the mean of the minority variants on the data on figure 3B. The results confirm our and the reviewer’s observation that the major variants were of lower replicative capacity (Wilcoxon matched pairs signed rank test p= 0.03). The new figure below has replaced figure 3C in the manuscript and is also shown below.

b. *It is also unclear as to why the authors only analyzed 7 of the 15 multivariant cases. The data would be stronger with a larger number of cases.*

The reviewer accurately points out that we did not clearly explain which TF Gag had been analysed in each graph. While TF Gag was cloned in 12 cases of multivariant infection, only 7 cases of multivariant infection yielded virus stocks with sufficient titers for use in the replicative capacity assay at the chosen MOI, in both the major and the minor variants. There was a total of 18 TF Gag variants from the 7 patients whose distribution between the patients is as shown in Figure 3B. The 5 cases where only one of the multiple TFs produced sufficient virus titers for use in our replicative capacity assay format are also now shown in figure 3B but these have not been included in the matched pairwise comparison pairwise on the new figure 3C as we do not know the replicative capacities of the other variants present within those patients. TF Gag from 3 patients could not be cloned as every cloning attempt yielded chimeric plasmids that did not express the patient derived TF Gag. This has been clarified in the manuscript and in figure 3B and marked in track changes within the main manuscript.

c. *Fig. 3C is also not accurate. The data points for the correlation are not independent (numerous data points are from the same individuals). An assumption for the statistical test (Spearman rank correlation) is that all data points are independent.*

In the original figure 3C, we had combined the RC data from the all clones from the all the patients from whom we cloned both major and minor variants (n of clones=18) with the 5 clones from the patients from whom we were only able to clone one of their multiple TFs. We agree with the noted lack of independence of the data as an assumption of the spearman rank correlation rest and have replaced this analysis with a new figure as suggested in reviewer 1’s comment 2a above, where we have categorised the variants as either major or minor variants, whereby the data in the minor variant column is a mean of the minor variants in the cases where there was more than one minor variant.

3. *Fig. 4 requires greater explanation. Which of the gag sequences were used in the multiple variants? Did it comprise only 1 variant from 12 individuals with multiple founders? If so, how and why was this 1 variant chosen? Are there multiple gag sequences from the same patients for the multiple variants’ column in this figure? If so, this would violate the assumption of independence for the statistical test. Should a weighted average (based on frequency of detection) of gag fitness from the individuals with multiple variants be used?*

The RC used for the multivariant infection column in figure 4 is the mean RC where more than 1 TF was cloned (n=7) and the RC of the cloned TF where only one RC was cloned (n=5). This has also been clarified in the manuscript.

4. *They also examined changes in T and B cells among individuals with multiple and single variants. This analysis is incomplete. There are only a limited number of individuals examined especially for those with multiple founders. Why was the analysis restricted to a small number of individuals? There are also multiple comparisons, and authors should acknowledge the possibility of the type 1 error due to examination of limited number of specific samples and multiple comparisons.*

The subset analysed is based on PBMC availability at the required time points. Analysis includes Benjamini-Hochberg correction for multiple comparisons, and this has been indicated in the “statistical analysis” section of the materials and methods section.

**Point-by-Point Response to Reviewer #2’s remarks:**

1. *The authors mention high risk behaviour and presence of STIs within the cohort. In addition, while epidermal barrier is primarily associated with increased risk of multivariant infection in MSM, presence of an existing STI is also noted as a possible determinant. Could the authors address the following?*
   a. *Can the authors comment on the presence of STIs potentially having an influence on the state of immune cell markers and differences in characteristics of CD4 T cell and B cell characteristics between the two groups? Can the authors account for presence or absence of an STI (or types of STIs if prevalent in both groups) and comment on this as a potential confounder, if this information exists?*

We would like to thank the reviewer for the excellent suggestion that we pursue further analysis on the presence and types of STI present in both single and multiple variant infection. STI testing in this cohort was mostly targeting symptomatic individuals and had shown that *Neisseria gonorrhoea* confirmed by gram staining was the strongest predictor of HIV acquisition increasing the risk of infection by up to 15-fold (10, 11). Of note, relatively high prevalence of asymptomatic STI has been shown in in this population(10, 12). We have now compared the rates of gram stain confirmed *Neisseria gonorrhoea* infection in individuals that were infected with multiple TF variants to those who were infected with one TF variant where *Neisseria gonorrhoea* infection was defined as the detection of gram-negative, intracellular diplococci consistent with *Neisseria gonorrhoea* in urethral or rectal secretions.

Out of the 38 MSM in this study, 15 were tested for gonococcal infection based on syndromic indication (7 with multivariant infection and 8 with single variant infection). 5 out of the 15 tested MSM had recent gonococcal infection within 180 days from infection. 4 of these 5 confirmed *Neisseria gonorrhoea* infections were infected with multiple HIV founder variants while only one out of five were infected with a single HIV founder variant (new figure 4B also shown below). Overall, 57% of the volunteers infected with multiple transmitted/founder variants and tested for *Neisseria gonorrhoea* (4 out of 7) had a recent *Neisseria gonorrhoea* infection compared to 12.5% (1 out of 8) of those who were infected with a single transmitted/founder variant and tested for *Neisseria gonorrhoea*. Despite the small sample size, these findings confirm our previous hypothesis that a breach of the genital mucosa resulting from recent STI infection was likely to be a risk factor for the acquisition of multiple transmitted/founder variants, which we show is linked to worse disease outcome.

Dissecting the role of recent *Neisseria gonorrhoea* and its treatment in the observed immune perturbances would require a comparison of the frequency of each cellular phenotype between patients infected with multiple transmitted/founder variants and had a recent *Neisseria gonorrhoea* infection to those infected with multiple transmitted/founder variants who did not have a recent *Neisseria gonorrhoea* infection. Due to the small sample size that had all the required measurements and the requirement to correct for multiple comparisons, we were not powered to perform this analysis. Nonetheless, this is an important question that should be pursued in a larger dataset. This has been discussed in the “discussion” part of the manuscript text and marked in track changes.

*b. Are data available pre-infection for a baseline indication of immune cell markers prior to HIV infection between those who acquired single versus multivariant infection?*

While we acknowledge that this information would be very useful, unfortunately the protocol C cohort from which these samples were obtained was a pre-infection incidence study and no PBMC were collected prior to infection.

*c. Could the authors comment as to whether any STI treatment was provided post HIV diagnosis and if so, could this potentially contribute to the observed improvement in immune perturbances?*

Treatment was provided for all symptomatic participants, but most *Neisseria gonorrhoea* infections are subclinical and norfloaxin and ciprofloxacine which were the recommended treatment for urethral or rectal discharge at the time are likely to have been ineffective due to documented resistance to these drugs in that cohort (10, 12). Participants will have therefore likely had repeated STI. Persisting inflammation of urethra or rectum due to unsuccessfully treated or undetected infections may have contributed to the increased risk of acquiring multivariant HIV infection and has been discussed in the discussion section of the manuscript text and marked in track changes.

*2. CTL escape can occur rapidly in acute infection and have particular impact on viral fitness when occurring in Gag. Can the authors comment as to a potential role of CTL escape in Gag in the difference in replication capacity of dominant versus minor variants in the multivariant infections? Were putative fitness-associated Gag mutations screened for in the clones utilized for the replication assays?*

CTL escape mutation within Gag early in infection is rare but when it does, it can influence viral replication in the newly infected patient and often adversely affects disease progression (13-16). Previous studies have shown that the RC of Gag-MJ4 chimeras correlated with the viral load of chronically infected donors near the estimated date of infection where rare mutations had the greatest effect on fitness (17, 18). In our selection of transmitted/founder *gag* genomes to clone into the chimeric viruses that were evaluated for replicative capacity, **we defined the transmitted/founder *gag* as having the least number of mutations and in all cases was the consensus sequence amongst each patients quasispecies.** Thus, it is likely that any CTL escape mutations that could be observed would have been transmitted from the source of the infection and not a result of *de novo* CTL escape. Furthermore, there was no evidence for increased diversity as demonstrated by average pairwise distance in variants that were sequences at the earliest timepoint (~14 days post infection) compared to those that were sequences later during acute infection (~60 days post infection) (Figure 1D), suggesting that the sequences were obtained prior to viral adaptation to the new host and were therefore close to the initial TF(s) and any escape variants are likely to have been inherited from the source of the infection.

Due to the known influence of such preadaptation to disease progression (13-16), we investigated whether the TF variants in the 7 cases where we could study both major and minor variants carried known HLA escape mutations that were associated with the patients HLA 1 alleles. A published reference database that contains information about sequence variants and mutations of HIV-1 CTL epitopes (LANLEpitope) was used. 5 out of the 7 patients each carried 1 known escape variant in Gag while 2 patients each carried 2 escape HLA associated mutations within Gag. However, the escape mutations were present in both the major and the minor variants, suggesting no role in the observed differences in replicative capacity between major and minor variants.

*3. Can the screening intervals for HIV infection be indicated in the methods?*

Of the 38 study participants 38 included, 27 were on monthly follow-up and 10 were in quarterly follow up at the HIV positive date. This has been clarified in the methods section and indicated in track changes.

*4. The authors introduce the relevance of the study as linked to prevention and cure of HIV, and later in the discussion section place emphasis on need for understanding pathogenesis. While all may apply, it would be good to provide a clearer description in terms of rationale and where this study specifically fits in.*

Our findings fit with HIV pathogenesis as we describe biological features of multivariant HIV infection that influence disease pathogenesis. However, HIV cure and treatment efforts benefit from a thorough understanding of HIV pathogenesis, especially the early events following HIV infection. Understanding HIV pathogenesis in relation to the transmission bottleneck can inform cure strategies as infection with multiple transmitted/founder variants is likely to influence the number of variants and complexity in the HIV reservoir, and therefore should be a consideration in the design of approaches to HIV treatment and cure. The relationship between HIV treatment and multivariant infection has also been recently highlighted by recent findings that the number of HIV-1 founder variants is determined by the recency of the source partner infection (19), which suggests that early diagnosis and initiation of treatment may provide a benefit in reducing the multiplicity of infection.

*5. In the discussion, the reduced bottleneck in MSM transmission is reported as though novel, while the authors do reference previous studies on this phenomenon. It would be of greater interest to compare the percentage single vs multi-variant infection in this study to that of previous studies, or the number of participants investigated, and to comment on any contrasts and factors that may have influenced differences between MSM cohort studies, if any.*

These points have been extensively discussed in the first comment addressed to reviewer 1.

**References**

1.	Chaillon A, Gianella S, Little SJ, Caballero G, Barin F, Kosakovsky Pond S, et al. Characterizing the multiplicity of HIV founder variants during sexual transmission among MSM. Virus Evol. 2016;2(1):vew012.

2.	Gottlieb GS, Heath L, Nickle DC, Wong KG, Leach SE, Jacobs B, et al. HIV-1 variation before seroconversion in men who have sex with men: analysis of acute/early HIV infection in the multicenter AIDS cohort study. J Infect Dis. 2008;197(7):1011-5.

3.	Janes H, Herbeck JT, Tovanabutra S, Thomas R, Frahm N, Duerr A, et al. HIV-1 infections with multiple founders are associated with higher viral loads than infections with single founders. Nat Med. 2015;21(10):1139-41.

4.	Keele BF, Giorgi EE, Salazar-Gonzalez JF, Decker JM, Pham KT, Salazar MG, et al. Identification and characterization of transmitted and early founder virus envelopes in primary HIV-1 infection. Proc Natl Acad Sci U S A. 2008;105(21):7552-7.

5.	Li H, Bar KJ, Wang S, Decker JM, Chen Y, Sun C, et al. High Multiplicity Infection by HIV-1 in Men Who Have Sex with Men. PLoS Pathog. 2010;6(5):e1000890.

6.	Rolland M, Tovanabutra S, deCamp AC, Frahm N, Gilbert PB, Sanders-Buell E, et al. Genetic impact of vaccination on breakthrough HIV-1 sequences from the STEP trial. Nat Med. 2011;17(3):366-71.

7.	Haaland RE, Hawkins PA, Salazar-Gonzalez J, Johnson A, Tichacek A, Karita E, et al. Inflammatory genital infections mitigate a severe genetic bottleneck in heterosexual transmission of subtype A and C HIV-1. PLoS Pathog. 2009;5(1):e1000274.

8.	Amornkul PN, Karita E, Kamali A, Rida WN, Sanders EJ, Lakhi S, et al. Disease progression by infecting HIV-1 subtype in a seroconverter cohort in sub-Saharan Africa. Aids. 2013;27(17):2775-86.

9.	Price MA, Rida W, Kilembe W, Karita E, Inambao M, Ruzagira E, et al. Control of the HIV-1 Load Varies by Viral Subtype in a Large Cohort of African Adults With Incident HIV-1 Infection. J Infect Dis. 2019;220(3):432-41.

10.	Duncan S, Thiong'o AN, Macharia M, Wamuyu L, Mwarumba S, Mvera B, et al. High prevalence of quinolone resistance in Neisseria gonorrhoeae in coastal Kenya. Sex Transm Infect. 2011;87(3):231.

11.	Sanders EJ, Okuku HS, Smith AD, Mwangome M, Wahome E, Fegan G, et al. High HIV-1 incidence, correlates of HIV-1 acquisition, and high viral loads following seroconversion among MSM. Aids. 2013;27(3):437-46.

12.	Sanders EJ, Thiong'o AN, Okuku HS, Mwambi J, Priddy F, Shafi J, et al. High prevalence of Chlamydia trachomatis and Neisseria gonorrhoeae infections among HIV-1 negative men who have sex with men in coastal Kenya. Sex Transm Infect. 2010;86(6):440-1.

13.	Brumme ZL, Brumme CJ, Carlson J, Streeck H, John M, Eichbaum Q, et al. Marked epitope- and allele-specific differences in rates of mutation in human immunodeficiency type 1 (HIV-1) Gag, Pol, and Nef cytotoxic T-lymphocyte epitopes in acute/early HIV-1 infection. J Virol. 2008;82(18):9216-27.

14.	Chopera DR, Ntale R, Ndabambi N, Garrett N, Gray CM, Matten D, et al. Early evolution of human leucocyte antigen-associated escape mutations in variable Gag proteins predicts CD4+ decline in HIV-1 subtype C-infected women. Aids. 2017;31(2):191-7.

15.	Martinez-Picado J, Prado JG, Fry EE, Pfafferott K, Leslie A, Chetty S, et al. Fitness cost of escape mutations in p24 Gag in association with control of human immunodeficiency virus type 1. J Virol. 2006;80(7):3617-23.

16.	Mónaco DC, Dilernia DA, Fiore-Gartland A, Yu T, Prince JL, Dennis KK, et al. Balance between transmitted HLA preadapted and nonassociated polymorphisms is a major determinant of HIV-1 disease progression. J Exp Med. 2016;213(10):2049-63.

17.	Prince JL, Claiborne DT, Carlson JM, Schaefer M, Yu T, Lahki S, et al. Role of transmitted Gag CTL polymorphisms in defining replicative capacity and early HIV-1 pathogenesis. PLoS Pathog. 2012;8(11):e1003041.

18.	Thompson RN, Wymant C, Spriggs RA, Raghwani J, Fraser C, Lythgoe KA. Link between the numbers of particles and variants founding new HIV-1 infections depends on the timing of transmission. Virus Evol. 2019;5(1):vey038.

19.	Villabona-Arenas CJ, Hall M, Lythgoe KA, Gaffney SG, Regoes RR, Hué S, et al. Number of HIV-1 founder variants is determined by the recency of the source partner infection. Science. 2020;369(6499):103-8.
